# Modifiable Parental Factors and Adolescent Sleep During Early Adolescence

**DOI:** 10.1001/jamanetworkopen.2025.31333

**Published:** 2025-09-11

**Authors:** Rosalind Ge, Sarah Whittle, Sarah P. H. Khor, Marie B. H. Yap, Bei Bei, Vanessa Cropley

**Affiliations:** 1 Centre for Youth Mental Health, University of Melbourne, Parkville, Australia; 2 Orygen, Parkville, Australia; 3 School of Psychological Sciences, Turner Institute for Brain and Mental Health, Monash University, Melbourne, Australia; 4 SEED Lifespan, School of Psychology, Deakin University, Melbourne, Australia

## Abstract

**Importance:**

Sleep problems are common in adolescence and are associated with poorer mental and physical health. Parental factors may be associated with adolescent sleep, providing potential targets for sleep health interventions. Whether these associations are mediated through emotional regulation and screen use and whether they vary by adolescent sex remain unclear.

**Objective:**

To investigate prospective associations between modifiable parental factors and sleep outcomes among adolescents 4 years later, with examination of the mediating roles of screen use and emotional regulation and the moderating role of adolescent sex.

**Design, Setting, and Participants:**

This preregistered prospective cohort study collected data from 3 waves (ages 9-11 [wave 1], ages 12-13 [wave 2], and ages 13-14 years [wave 3]) in the US-based Adolescent Brain Cognitive Development (ABCD) Study from June 1, 2016, to October 15, 2018. Data were analyzed from February 20 to November 13, 2024. Participants include a nationally representative sample of 3419 youths with sleep data available at wave 3.

**Exposures:**

Parental factors, mediating variables (emotional regulation and screen use), and the moderator variable (adolescent sex).

**Main Outcomes and Measures:**

Adolescent duration, timing, chronotype, quality, and regularity of sleep were assessed using actigraphy or questionnaires at wave 3.

**Results:**

Among 3419 adolescents included in the analysis (1806 [52.8%] male; mean [SD] age, 9.49 [0.50] years), greater parental monitoring was associated with better sleep quality (β = 0.04; 95% CI, 0.01-0.07; *P* = .04). Higher family conflict was associated with later chronotype (β = 0.04; 95% CI, 0.01-0.08; *P* = .04) and poorer sleep quality (β = −0.04; 95% CI, −0.07 to −0.01; *P* = .04). Parental psychopathology was associated with later sleep timing (β = 0.06; 95% CI, 0.02-0.11; *P* = .03), later chronotype (β = 0.06; 95% CI, 0.03-0.09; *P* = .01), and poorer sleep quality (β = −0.29; 95% CI, −0.31 to −0.26; *P* < .001). Adolescent screen use partially mediated most of these associations, while the mediating role of emotional regulation was limited to sleep quality. Sex moderated the association between parental warmth and earlier chronotype in girls (β = −0.14; 95% CI, −0.22 to −0.06; *P* = .007).

**Conclusions and Relevance:**

In this prospective study of the ABCD cohort, modifiable parental factors in early adolescence were associated with specific adolescent sleep outcomes 4 years later, with screen use and emotional regulation serving as mediators. Adolescent sex moderated the association between parental warmth and sleep chronotype. These findings highlight potential targets for evidence-based interventions to improve adolescent sleep health.

## Key Points

**Question:**

How are modifiable parental factors when children are 9 to 11 years of age associated with adolescent sleep at 13 to 14 years of age, and what are the mediating and moderating factors?

**Findings:**

In this cohort study of 3419 adolescents, family conflict and parental psychopathology were associated with later sleep timing, later chronotype, and poorer sleep quality, whereas parental monitoring was associated with better sleep quality and parental warmth with earlier chronotype in girls. Screen use mediated associations with sleep timing and chronotype, while emotional regulation mediated the association with sleep quality.

**Meaning:**

These findings suggest that sex-informed, family-based interventions may improve adolescent sleep health.

## Teaser

This cohort study examines the associations between parental factors when children are 9 to 11 years of age and multidimensional sleep outcomes at 13 to 14 years of age, with the mediating roles of emotional regulation and screen use and the moderating role of sex.

## Introduction

Early adolescence is marked by significant neurobiological, hormonal, and sociocontextual changes that impact sleep health.^1,2^ Sleep problems, including difficulties initiating and maintaining sleep, delayed sleep timing, and irregular sleep-wake patterns, are prevalent during adolescence^3,4,5^ and associated with adverse outcomes such as lower academic and/or cognitive performance,^6,7^ emotional dysregulation,^8^ and compromised physical growth.^9,10^ Identifying modifiable factors promoting healthy adolescent sleep is therefore crucial for developing targeted interventions.

Drawing on ecological systems theory by Bronfenbrenner,^11^ which highlights the central role of the family environment and its interactions with broader environmental systems in shaping developmental outcomes, we focused on modifiable parental factors, which may play an important role in adolescent sleep.^12,13^ A 2021 systematic review and meta-analysis^14^ defined modifiable parental factors as “behavioural or cognitive factors within the capacity of parents to alter, through self-directed action or psychological intervention,” and identified parental warmth, parental monitoring, family conflict, and parental psychopathology as having emerging evidence of association with adolescent sleep. However, as discussed by the authors, most research has been cross-sectional, assessed limited parenting and sleep domains, and relied solely on self-reported measures of sleep. Prospective studies incorporating these modifiable parental factors with both objective and subjective sleep measures are needed to verify the associations and identify therapeutic targets.

The adapted bioecological model of Bronfenbrenner emphasizes the importance of proximal processes—the ongoing, reciprocal interactions between adolescents and their immediate environment—as primary engines of development. Within this framework,^15^ we conceptualize emotional regulation and screen use as important mediators linking parental factors to adolescent sleep. Parenting practices can directly shape emotional regulation skills, which are essential for healthy sleep patterns through their effects on stress management and bedtime routines.^16,17,18^ Likewise, parental factors influence adolescents’ screen use behaviors,^19,20^ which in turn impact sleep through mechanisms such as circadian rhythm disruption and increased cognitive arousal.^21^ Understanding these mediating pathways is critical for designing targeted, scalable interventions.

Additionally, the Bronfenbrenner model accommodates exploration of moderators such as sex, as empirical evidence supports sex-specific responses to parenting and differences in sleep patterns during adolescence. Some studies suggest girls may be more sensitive to parental warmth and family climate, while males may be more affected by parental monitoring or discipline.^22,23^ However, prospective examinations of such mediators and moderators during early to middle adolescence remain absent.

The present study addressed these gaps by using a large longitudinal sample of youths from the Adolescent Brain and Cognitive Development (ABCD) Study to: (1) examine the prospective associations between parental factors (warmth, monitoring, conflict, and psychopathology) at 9 to 11 years of age and multidimensional sleep outcomes (duration, timing, chronotype, quality, and regularity) at 13 to 14 years of age, (2) test the mediating roles of emotional regulation and screen use, and (3) explore the moderating role of adolescent sex. The selection of parental factors is guided by the framework of Khor et al,^14^ while sleep outcomes are informed by the Buysse multidimensional sleep health model.^24^ We hypothesized that greater parental warmth and monitoring would be associated with favorable sleep outcomes, while higher family conflict and parental psychopathology would be linked to less favorable sleep outcomes, with screen use and emotional regulation serving as partial mediators. Given limited prior evidence, no prior hypotheses were proposed regarding sex differences.

## Methods

Data for this cohort study were drawn from the ABCD study, data release 5.1, from June 1, 2016, to October 15, 2018. Full sampling, recruitment, protocol, and procedures are available online.^25^ The study was preregistered on the Open Science Framework.^26^ Participants provided assent and caregivers provided written informed consent to protocols approved by institutional review boards at each data collection site. The study followed the Strengthening the Reporting of Observational Studies in Epidemiology (STROBE) reporting guideline.

### Participants

This study analyzed data from 3 waves (9-11 years of age [wave 1], 12-13 years of age [wave 2], and 13-14 years of age [wave 3]), with parental factors examined at wave 1, mediators at wave 2, and sleep outcomes at wave 3. Participants with missing sleep outcomes at wave 3 were excluded. To address familial dependency, one sibling per family was randomly selected. The analytic sample size for each model ranged from a minimum of 571 to a maximum of 3419 participants, depending on the number of participants with complete data for the specific combination of sleep outcome and parental factors included in each analysis. A comparison of sociodemographic characteristics between included and excluded participants is presented in eTable 1 in Supplement 1, and site-specific descriptive statistics for the ABCD cohort at baseline are provided in eTable 2 in Supplement 1.

### Measures

#### Modifiable Parental Factors (Wave 1)

A full description of study measures is provided in the eMethods in Supplement 1. Parental warmth was assessed using the youth-reported Acceptance subscale of the Child Report of Parent Behavior Inventory.^27,28,29^ Parental monitoring was measured using 5 items from the youth-reported Parental Monitoring Survey.^30^ Family conflict was evaluated using the youth-reported Family Conflict subscale of the Moos Family Environment Scale.^31^ Parental psychopathology was assessed using the parent-reported Adult Self Report.^32,33^ The total score was computed for each respective scale.

#### Sleep Outcomes (Wave 3)

Sleep was assessed using both actigraphy and parent-reported questionnaires. Actigraphy data (Fitbit Charge HR; Google Inc) provided measures of sleep duration, timing, and regularity. Sleep duration was operationalized as the mean nightly total sleep time (in minutes), while sleep timing was defined as the mean sleep midpoint (in hours from midnight). These metrics were calculated using a weighted mean of weekday and weekend recordings, following established methodology.^34^ Participants with a minimum of 7 nights of valid actigraphy data during the 3-week period were included.^35,36^ Sleep regularity was assessed using the Sleep Regularity Index for participants with a minimum of 7 consecutive days of actigraphy data.^37^ The youth-reported Munich Chronotype Questionnaire^38,39^ assessed chronotype, with higher values representing an evening-type chronotype. The parent-reported Sleep Disturbance Scale for Children^40^ (reversed scored by multiplying by −1) evaluated sleep quality, with higher scores indicating better overall sleep quality.

#### Mediators (Wave 2)

Screen use was assessed using the youth-reported Youth Screen Time Survey^41^ as total screen time spent on typical days. A total daily screen use was calculated as a weighted mean across weekdays and weekends with the following formula: ([weekday hours ×5] + [weekend hours ×2])/7.^42^ Parent-reported adolescent emotion regulation was assessed using the reverse-scored Difficulties in Emotion Regulation Scale,^43^ with higher scores indicating better emotion regulation.

#### Covariates (Wave 1)

We covaried for age, pubertal status, socioeconomic status (SES), and sex (assigned at birth) in all models. Pubertal status was measured using the Pubertal Development Scale,^44^ and SES was estimated using the income-to-needs ratio, calculated by dividing the parent-reported total household income by the federal poverty threshold for the given household size^45^ (eMethods in Supplement 1).

### Statistical Analysis

Data were analyzed from February 20 to November 13, 2024. Detailed data preparation and statistical analysis can be found in the eMethods in Supplement 1. Variables at or above 3.5 SDs were winsorized to mitigate the impact of extreme outliers and standardized (*z* scored) to allow for direct comparison of effect sizes. Missing data were primarily handled using pairwise deletion, and mean imputation was used for parental factors with sporadic missingness. Full details and sensitivity analyses using multiple imputations are reported in the eMethods in Supplement 1.

We used linear mixed models to examine the association between parental factors at wave 1 and sleep outcomes at wave 3, accounting for covariates and the nested data structure (participants within research sites). Site was included as a random effect. Statistical significance was determined using a 2-sided *P* < .05. To correct for multiple comparisons, the Benjamini-Hochberg false discovery rate (FDR)^46^ was applied across the set of models (4 parental factors ×5 sleep outcomes). Reported *P* values are corrected for FDR.

For significant parent-adolescent sleep associations, mediation analyses were conducted using path analysis to assess the mediation (indirect) role of emotional regulation and screen use with the association between the parental factor and sleep outcome. We specified 1000 simulations to estimate each mediating role and the 95% CI using quasi-bayesian Monte Carlo approximation.^47^

To examine whether parent-adolescent sleep associations differed by sex assigned at birth, moderation analyses were performed by adding an interaction term between sex and each parental factor to the linear mixed models, applying the same FDR correction as described above. For models with significant sex-by-parental factor interaction outcomes, a simple slopes analysis was performed, as well as mediation analyses of screen use and emotional regulation for each sex separately.

We conducted 2 preregistered sensitivity analyses. First, given the association between race and SES,^48^ race and ethnicity were not included as a covariate in primary analyses but were added in sensitivity analyses. Second, results from pairwise deletion were compared with those using multiple imputations (eMethods in Supplement 1).

## Results

Participant characteristics of the maximum analytic sample (N = 3419) after excluding participants with missing year 4 sleep data are presented in Table. Mean (SD) age was 9.49 (0.50) years. A total of 1613 participants (47.2%) were female and 1806 (52.8%) were male.

**Table.** Participant Characteristics

Variable	Participants (N = 3419)^a^
Age, mean (SD), y	9.49 (0.50)
Biological sex, No. (%)	
Female	1613 (47.2)
Male	1806 (52.8)
Race and ethnicity, No. (%)^b^	
Asian	87 (2.5)
Black	323 (9.4)
Hispanic	689 (20.0)
White	1983 (58.0)
Other race^c^	337 (9.9)
Pubertal status, mean (SD)^d^	1.75 (0.85)
Income-to-needs ratio, mean (SD)^e^	4.17 (2.88)
Sleep outcomes at 4-y follow-up, mean (SD)	
Sleep duration, min (n = 1161)^f^	423.57 (41.24)
Sleep timing, h after midnight (n = 1161)^f^	3.95 (1.76)
Sleep chronotype (n = 3008)^g^	28.45 (1.75)
Sleep quality^h^	35.90 (7.73)
Sleep Regularity Index (n = 571)^i^	36.19 (16.76)
Parental factors at baseline, mean (SD)	
Parental warmth^j^	13.92 (1.51)
Parental monitoring^k^	22.03 (2.48)
Family conflict^l^	1.94 (1.91)
Parental psychopathology^m^	424.90 (28.92)
Mediators at 3-y follow-up, mean (SD)	
Emotion regulation (n = 3402)^n^	66.57 (13.92)
Screen use, h (n = 3379)	2.60 (2.17)

ªSample sizes vary across analyses due to missing data. The eMethods in Supplement 1 gives details on participant selection and data inclusion.

^b^ Collected by the parent report.

^c^ American Indian or Alaska Native, Native Hawaiian or Other Pacific Islander (eg, Samoan, Guamanian), multiracial, or declined to answer.

^d^ Measured using the Pubertal Development Scale. Scores range from 1 to 4, with higher scores indicating more advanced pubertal stage.

^e^ Calculated following established methods of Gonzalez et al.^45^

^f^ Calculated by dividing the parent-reported total household income by the federal poverty threshold for the given household size.

^g^ Measured using the Munich Chronotype Questionnaire. The resulting chronotype score, midsleep on free days, reflects the individual’s sleep phase preference, with higher values representing a delayed, evening-type chronotype. The score ranges from 16 to 40, with each unit corresponding to 1 hour (eg, 26.33 = 2:20 am).

^h^ Measured by total scores in the parent-reported Sleep Disturbance Scale for Children. Scores range from 26 to 130, with higher scores indicating poorer overall sleep quality.

^i^ Calculated using epoch-level sleep-wake data. Details of the algorithm are given in the package documentation and Windred et al.^37^ Scores range from 0 to 100, with higher scores reflecting greater sleep regularity.

^j^ Measured using the youth-reported Acceptance subscale of the Child Report of Parent Behavior Inventory. Scores range from 5 to 15, with higher scores indicating greater levels of parental warmth experienced by youths.

^k^ Measured using the youth-reported Parental Monitoring Survey. Scores range from 5 to 25, with higher scores indicating greater levels of perceived parental monitoring.

^l^ Measured using the youth-reported Family Conflict subscale of the Moos Family Environment Scale. Scores range from 0 to 9, with higher scores indicating greater levels of perceived family conflict.

^m^ Measured using the parent-reported Adult Self Report. Scores range from 400 to 780, with higher scores indicating greater levels of psychopathology experienced by the parent.

^n^ Measured using the parent-reported Difficulties in Emotion Regulation Scale (DERS). Scores range from 36 to 180, with higher scores indicating greater difficulties with emotion regulation.

### Association Between Parental Factors and Sleep Outcomes

**Figure 1.**
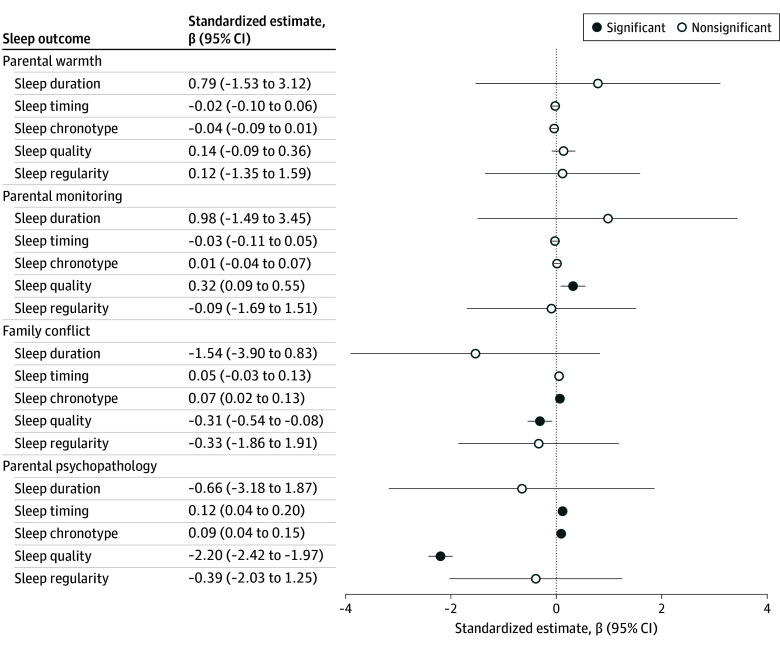
Associations Between Parental Factors and Sleep Outcomes Standardized parameter estimates (β) and 95% CIs from linear mixed models depicting the associations between parental factors (parental warmth, parental monitoring, family conflict, and parental psychopathology) measured at baseline and 5 sleep outcomes (sleep duration, timing, chronotype, quality, and regularity) measured 4 years later. All models were controlled for age, sex, pubertal status, and income-to-needs ratio. Site was modeled as a random effect. The dashed vertical line represents the null effect (β = 0).

Parental warmth was not associated with any sleep outcome. Higher parental monitoring showed a positive association with sleep quality (β = 0.04; 95% CI, 0.01-0.07; *P* = .04). Higher family conflict was associated with later chronotype (β = 0.04; 95% CI, 0.01-0.08; *P* = .04) and poorer sleep quality (β = −0.04; 95% CI, −0.07 to −0.01; *P* = .04). Parental psychopathology was associated with later sleep timing (β = 0.06; 95% CI, 0.02-0.11; *P* = .03), later chronotype (β = 0.06; 95% CI, 0.03-0.09; *P* = .01), and poorer sleep quality (β = −0.29; 95% CI, −0.31 to −0.26; *P* < .001) (Figure 1).

### Mediation Analysis

**Figure 2.**
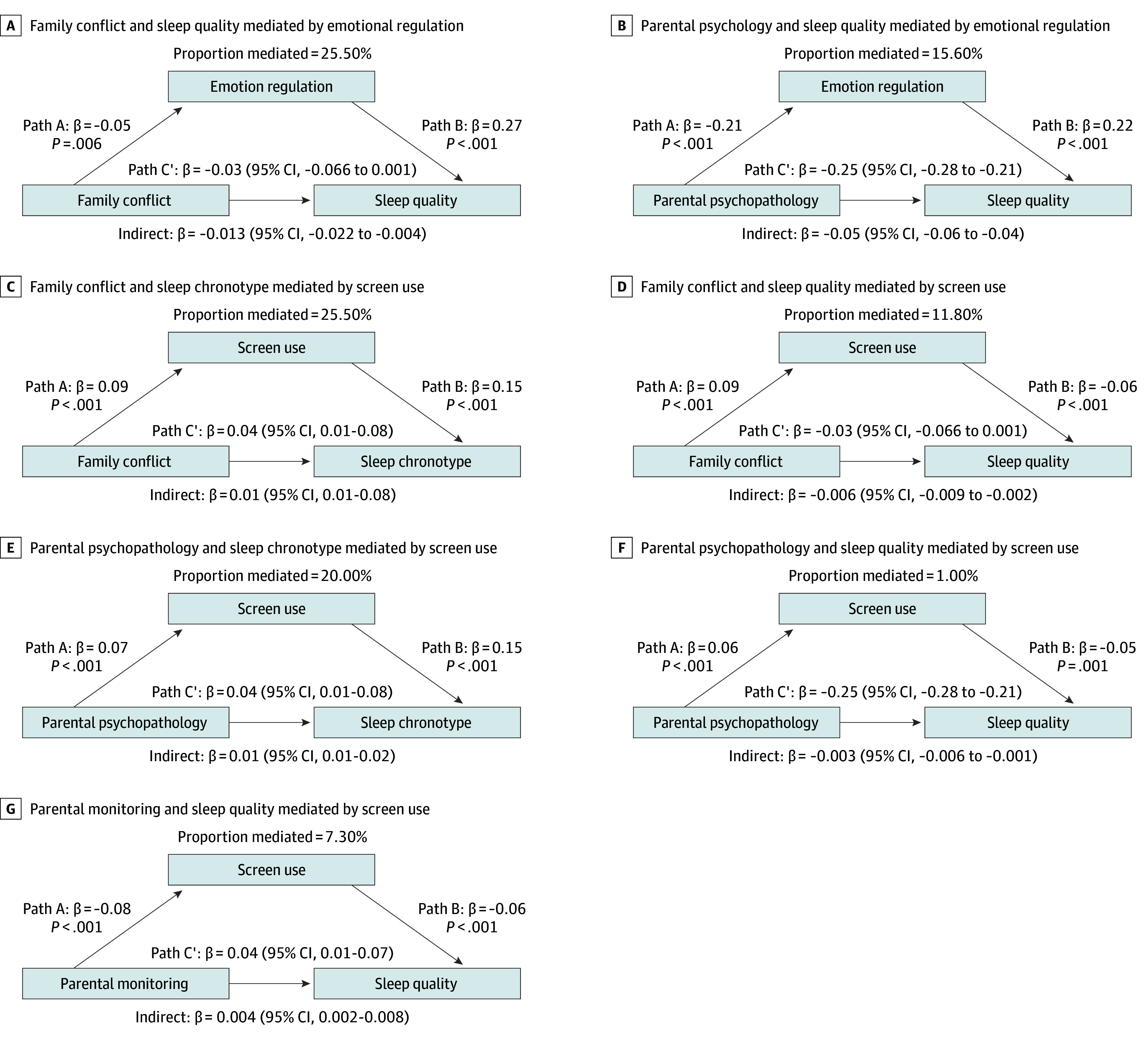
Mediation Models for Association of Parental Factors With Adolescent Sleep Outcomes Through Emotional Regulation and Screen Use Results from mediation analyses examining the indirect association of parental factors with adolescent sleep outcomes, with emotion regulation (A and B) and screen use (C-G) tested as separate mediators (both measured at 3-year follow-up). Each panel shows the proportion of the effect mediated, along with standardized coefficients (β) and *P* values for paths A and B. The direct effect (C’) and indirect effect size, with corresponding 95% CIs are also presented. Significant indirect effect is indicated by bias-corrected bootstrap CIs that do not contain zero.

Significant models are shown in Figure 2. The indirect association of parental monitoring with better sleep quality was mediated through decreased screen use (β = 0.004; 95% CI, 0.002-0.008; proportion mediated [PM], 7.3%). The indirect association mediated through increased screen use was present for both family conflict (β = 0.01; 95% CI, 0.01-0.08; PM, 25.5%) and parental psychopathology (β = 0.01; 95% CI, 0.01-0.02; PM, 20.0%) to later chronotype. The indirect association of family conflict with poorer sleep quality was mediated through both worse emotional regulation skills (β = −0.01; 95% CI, −0.02 to −0.004; PM, 25.5%) and increased screen use (β = −0.003; 95% CI, −0.006 to −0.001; PM, 11.8%). Similarly, the indirect association of parental psychopathology with poorer sleep quality was mediated through both worse emotional regulation skills (β = −0.05; 95% CI, −0.06 to −0.04; PM, 15.6%) and increased screen use (β = −0.003; 95% CI, −0.01 to −0.001; PM, 1.0%).

### Moderation Analysis

**Figure 3.**
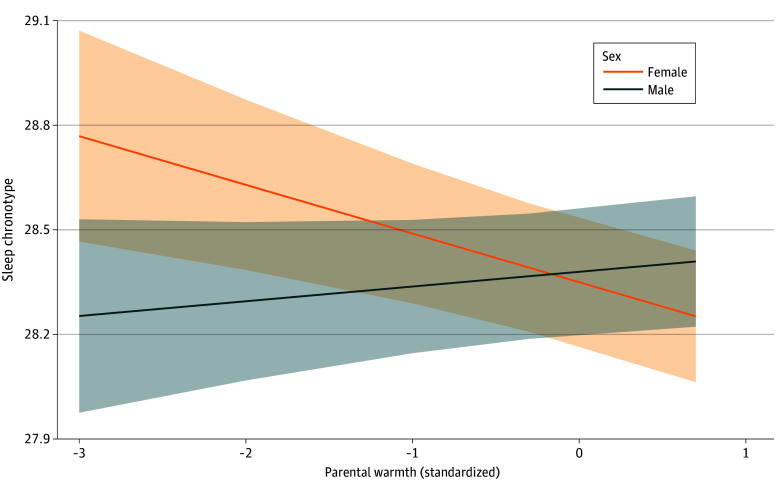
Sex-Specific Associations Between Parental Warmth and Adolescent Sleep Chronotype Simple slopes illustrate the association between standardized levels of parental warmth (range, –3.0 to 0.7, with higher indicating higher level of warmth) and adolescent sleep chronotype (range, 27.98-29.07, with higher indicating later chronotype), stratified by sex. Higher levels of parental warmth were associated with an earlier sleep chronotype for female adolescents but not for male adolescents. The shaded regions represent 95% confidence intervals around the simple slopes.

Adolescent sex interacted with parental warmth (β = 0.13; 95% CI, 0.06-0.19; *P* < .001) in association with sleep chronotype (Figure 3). Sex moderated the association of higher parental warmth with earlier chronotype in girls (β = −0.14; 95% CI, −0.22 to −0.06; *P* = .007), but not boys (β = −0.04; 95% CI, −0.03 to 0.11; *P* = .22). Given this interaction, mediation analysis was conducted separately for girls (stratified analysis) to investigate whether emotion regulation or screen use mediated the effects of parental warmth on sleep chronotype. No indirect association was found through mediation of either emotional regulation (95% CI, −0.01 to 0.002) or screen use (95% CI, −0.10 to 0.02). Full results are reported in eTable 3 in Supplement 1.

### Sensitivity Analysis

The inclusion of race and ethnicity as a covariate did not appreciably alter the findings. Results from the multiple imputation analysis for missing data were consistent with results of the primary pairwise analyses. However, the moderating role of sex in the association between parental warmth and chronotype did not remain significant. Detailed results are available in eFigures 1 and 2 and eTables 3 to 11 in Supplement 1.

## Discussion

Following previous recommendations and consistent with the Bronfenbrenner ecological systems theory,^11,14^ this cohort study prospectively examined modifiable parental factors associated with multidimensional constructs of adolescent sleep. Specifically, higher parental monitoring was associated with better sleep quality, while greater family conflict and parental psychopathology were associated with poorer sleep quality, later timing, and chronotype. Extending prior work,^49,50^ we also found support for screen use and emotion regulation as partial mediators of these associations. Notably, we observed a sex-specific association between parental warmth and chronotype among girls.

Among the positive parental factors examined, parental monitoring was associated with better adolescent sleep quality. Adolescents who perceived higher levels of parental monitoring were reported by their parents to have better sleep, underscoring the actionable role of parental factors. Parents who are attentive to their adolescents’ routines may help establish consistent bedtime,^51^ enforce healthy sleep habits, and limit activities—such as excessive screen time—that disrupt sleep patterns.^52,53^ In contrast, negative parental factors such as family conflict and parental psychopathology were associated with poorer sleep, including later chronotype and sleep timing and lower sleep quality. These findings align with an existing theory that a stressful or unsupportive family environment can undermine adolescent adjustment and sleep,^54,55^ potentially through increased emotional insecurity and chronic stress.^54,56,57^ Notably, the strongest association was observed between parent psychopathology and sleep quality. While this may be partially inflated due to a shared informant, it may also reflect the multifaceted nature of sleep quality that captures a broader range of sleep-related issues compared with discrete objective measures such as duration and regularity. This suggests that parental factors may exert cumulative influences on multiple sleep dimensions that objective measures alone may not fully capture.

A notable finding was the sex-specific association between parental warmth and earlier chronotype in female adolescents only. This finding may be explained by the heightened emotional sensitivity and stress reactivity of girls during puberty,^58^ making them more responsive to supportive family environments. Socialization practices, whereby girls may receive more warmth and protectiveness^22^ while boys are encouraged toward independence^23^ could also contribute to these differences. However, sensitivity analysis using imputation methods did not replicate this sex-specific association, warranting cautious interpretation. Nevertheless, our findings suggest that parental influences on chronotype may differ by sex, warranting further investigation into sex-specific mechanisms such as hormonal changes, gender roles, and maternal and paternal differences in parenting practices.

Not all sleep domains or parental factors were equally affected. Beyond the sex-specific chronotype finding, we observed no significant associations between parental warmth and other sleep outcomes. This may reflect the complex nature of the parental warmth–sleep association, which is influenced by contextual factors^59^ and informant perspectives. Our study used adolescents’ appraisal of parental warmth, unlike some previous research that relied on parent reports,^60^ and these perspectives may not always align. Similarly, we found no associations between parental factors and sleep duration^27,51,55,56,57,58^ or regularity.^61,62^ This discrepancy may stem from our use of actigraphy-derived measures rather than the subjective reports commonly used in prior studies, as these often capture different sleep constructs.^63^ The wide CIs in these estimates demonstrate considerable heterogeneity in how parental factors are associated with these sleep domains, possibly reflecting effects beyond parental influence, such as genetics^64^ and school schedules.^65^ Future studies should integrate both actigraphy and subjective reports to disentangle these complex relationships.

Consistent with our hypothesis, emotion regulation and screen use partially mediated associations between parental factors and sleep. Emotional regulation partially explained pathways between family conflict and parental psychopathology and sleep quality, consistent with the bioecological theory highlighting how parenting practices shape children’s self-regulation skills that influence health outcomes such as sleep.^18^ Screen use emerged as a particularly important mediator for the association of family conflict and parental psychopathology with both sleep quality and chronotype and of parental monitoring with sleep quality. Adolescents in high-conflict households might turn to screens to escape family stress or manage negative emotions. While such behavior may offer temporary relief, excessive screen use can disrupt sleep by delaying bedtime and increasing physiological and cognitive arousal.^66^ However, given that this study investigated total screen time only, future research is needed to identify which aspects of screen use, such as timing and type, contribute most strongly to sleep disruption and underpin these associations.

Our study has important implications. In line with the Bronfenbrenner ecological systems theory, our findings highlight how parental factors and broader family dynamics shape adolescent sleep outcomes through multiple interacting pathways. Specifically, our results underscore the value of routinely assessing parental mental health and family dynamics when evaluating adolescent sleep disturbances. Parenting strategies that encourage age-appropriate monitoring of adolescents, reduce family conflict, and address parental emotional distress may be especially useful for improving adolescent sleep quality and circadian timing. Evidence-based interventions such as the Family Check-Up^67^ and the Strengthening Families Program,^68^ can effectively improve parental monitoring and reduce family conflict within a brief time frame. Additionally, cognitive behavioral therapy for insomnia offers established sleep-specific strategies that can be delivered in individual, group, or digital formats.^69^ Importantly, our study also identified potential mechanisms (eg, screen use) that could be targeted in interventions to improve adolescent sleep. Furthermore, our findings highlight the potential value of tailoring interventions by sex, as girls may respond differently to certain parental factors, such as parental warmth. These multilevel approaches provide practical and scalable options for addressing both family dynamics and individual adolescent needs to promote healthier sleep outcomes.^70^ Although the standardized effect sizes observed in this study were small, consistent with other large-scale ABCD studies, these can have meaningful public health implications at the population level or in cumulative risk models.^71^

### Limitations

While our study benefits from a large sample size, prospective design, and use of both actigraphy-derived and subjective sleep measures, some limitations warrant consideration. First, the observational design precludes causal inferences. Randomized clinical trials targeting parental factors are needed to establish causality with adolescent sleep. Second, single time point assessments may not capture the dynamic nature of parent-child relationships. Future studies should incorporate repeated assessments from multiple informants to examine longitudinal and bidirectional associations. Third, actigraphy data were available for only a subset of participants at 4-year follow-up, and the absence of baseline actigraphy measures limited our ability to account for prior sleep problems, and individuals with insomnia were not excluded. Fourth, the inability to distinguish between vacation and school periods precluded analysis of social jet lag, a key feature of adolescent sleep patterns. Fifth, whereas this study is among the few to examine putative mechanisms and moderators of the parent-adolescent sleep association, future research should explore additional pathways, such as family or individual stress, SES, and pubertal timing to identify subgroups of adolescents particularly susceptible to sleep disruption in adverse family environments. Finally, our analytic approach to race and SES, detailed in the eMethods in Supplement 1, recognizes these as intersecting structural dimensions of inequity.^72^ Future studies should apply intersectional frameworks to further clarify the joint impact of these factors on adolescent sleep.

## Conclusions

In this prospective cohort study, modifiable parental factors in early adolescence were associated with specific adolescent sleep outcomes 4 years later. Family conflict and parental psychopathology were associated with poorer sleep quality, delayed sleep timing, and evening-type chronotype, while parental monitoring emerged as a protective factor for sleep quality. Screen use and emotional regulation partially mediated the associations between parental factors and sleep outcomes, highlighting their potential mechanistic roles in adolescent sleep patterns. The observed associations between parental warmth and chronotype in girls suggested the value of sex-specific interventions. Recognition of the pathways through which parental factors influence adolescent sleep may inform the development of targeted, evidence-based strategies to promote healthy sleep in adolescents.

## Notes

Supplement 1. **eMethods.** Measures, Mediators, and Statistical Analysis **eFigure 1.** Associations Between Parental Factors and Sleep Outcomes, Adjusted for Race (Sensitivity Analysis 1) **eFigure 2.** Associations Between Parental Factors and Sleep Outcomes Using Imputation (Sensitivity Analysis 2) **eTable 1.** Comparison of Included vs Excluded Participants on Key Demographics at Baseline **eTable 2.** Site-Specific Descriptive Statistics for the ABCD Cohort at Baseline **eTable 3.** Moderation Role of Adolescent Sex on the Associations Between Parental Factors and Sleep Outcomes **eTable 4.** Standardized Path Estimates of Parental Factors on Sleep Quality Mediated by Emotion Regulation and Screen Use, Including Race and Ethnicity as a Covariate (Sensitivity Analysis 1) **eTable 5.** Standardized Path Estimates of Parental Factors on Sleep Timing Mediated by Emotion Regulation and Screen Use, Including Race and Ethnicity as a Covariate (Sensitivity Analysis 1) **eTable 6.** Standardized Path Estimates of Parental Factors on Sleep Chronotype Mediated by Emotion Regulation and Screen Use, Including Race and Ethnicity as a Covariate (Sensitivity Analysis 1) **eTable 7.** Moderation Effects of Adolescent Sex: Associations Between Parental Factors and Sleep Outcomes, Including Race and Ethnicity as a Covariate (Sensitivity Analysis 1) **eTable 8.** Standardized Path Estimates of Parental Factors on Sleep Quality Mediated by Emotion Regulation and Screen Use, Using Multiple Imputation (Sensitivity Analysis 2) **eTable 9.** Standardized Path Estimates of Parental Factors on Sleep Timing Mediated by Emotion Regulation and Screen Use, Using Multiple Imputation (Sensitivity Analysis 2) **eTable 10.** Standardized Path Estimates of Parental Factors on Sleep Chronotype Mediated by Emotion Regulation and Screen Use, Using Multiple Imputation (Sensitivity Analysis 2) **eTable 11.** Moderation Effects of Adolescent Sex: Associations Between Parental Factors and Sleep Outcomes, Using Multiple Imputation (Sensitivity Analysis 2)
eReferences.

Supplement 2.
Data Sharing Statement
