# Supplementary material for: Modifiable Parental Factors and Adolescent Sleep During Early Adolescence
Source: JAMA Netw Open. 2025 Sep 11;8(9):e2531333. doi: 10.1001/jamanetworkopen.2025.31333 (PMC12426799; doi:10.1001/jamanetworkopen.2025.31333)
Supplement: Supplement 1. — eMethods. Measures, Mediators, and Statistical Analysis eFigure 1. Associations Between Parental Factors and Sleep Outcomes, Adjusted for Race (Sensitivity Analysis 1) eFigure 2. Associations Between Parental Factors and Sleep Outcomes Using Imputation (Sensitivity Analysis 2) eTable 1. Comparison of Included vs Excluded Participants on Key Demographics at Baseline eTable 2. Site-Specific Descriptive Statistics for the ABCD Cohort at Baseline eTable 3. Moderation Role of Adolescent Sex on the Associations Between Parental Factors and Sleep Outcomes eTable 4. Standardized Path Estimates of Parental Factors on Sleep Quality Mediated by Emotion Regulation and Screen Use, Including Race and Ethnicity as a Covariate (Sensitivity Analysis 1) eTable 5. Standardized Path Estimates of Parental Factors on Sleep Timing Mediated by Emotion Regulation and Screen Use, Including Race and Ethnicity as a Covariate (Sensitivity Analysis 1) eTable 6. Standardized Path Estimates of Parental Factors on Sleep Chronotype Mediated by Emotion Regulation and Screen Use, Including Race and Ethnicity as a Covariate (Sensitivity Analysis 1) eTable 7. Moderation Effects of Adolescent Sex: Associations Between Parental Factors and Sleep Outcomes, Including Race and Ethnicity as a Covariate (Sensitivity Analysis 1) eTable 8. Standardized Path Estimates of Parental Factors on Sleep Quality Mediated by Emotion Regulation and Screen Use, Using Multiple Imputation (Sensitivity Analysis 2) eTable 9. Standardized Path Estimates of Parental Factors on Sleep Timing Mediated by Emotion Regulation and Screen Use, Using Multiple Imputation (Sensitivity Analysis 2) eTable 10. Standardized Path Estimates of Parental Factors on Sleep Chronotype Mediated by Emotion Regulation and Screen Use, Using Multiple Imputation (Sensitivity Analysis 2) eTable 11. Moderation Effects of Adolescent Sex: Associations Between Parental Factors and Sleep Outcomes, Using Multiple Imputation (Sensitivity Analysis 2) eReferences. [file jamanetwopen-e2531333-s001.pdf]

Supplementary Online Content

Ge R, Whittle S, Khor SPH, Yap MBH, Bei B, Cropley V. Modifiable parental factors and adolescent sleep during early adolescence. *JAMA Netw Open.* 2025;8(9):e2531333. doi:10.1001/jamanetworkopen.2025.31333

eMethods. Measures, Mediators, and Statistical Analysis

eFigure 1. Associations Between Parental Factors and Sleep Outcomes, Adjusted for Race (Sensitivity Analysis 1)

eFigure 2. Associations Between Parental Factors and Sleep Outcomes Using Imputation (Sensitivity Analysis 2)

eTable 1. Comparison of Included vs Excluded Participants on Key Demographics at Baseline

eTable 2. Site-Specific Descriptive Statistics for the ABCD Cohort at Baseline

eTable 3. Moderation Role of Adolescent Sex on the Associations Between Parental Factors and Sleep Outcomes

eTable 4. Standardized Path Estimates of Parental Factors on Sleep Quality Mediated by Emotion Regulation and Screen Use, Including Race and Ethnicity as a Covariate (Sensitivity Analysis 1)

eTable 5. Standardized Path Estimates of Parental Factors on Sleep Timing Mediated by Emotion Regulation and Screen Use, Including Race and Ethnicity as a Covariate (Sensitivity Analysis 1)

eTable 6. Standardized Path Estimates of Parental Factors on Sleep Chronotype Mediated by Emotion Regulation and Screen Use, Including Race and Ethnicity as a Covariate (Sensitivity Analysis 1)

eTable 7. Moderation Effects of Adolescent Sex: Associations Between Parental Factors and Sleep Outcomes, Including Race and Ethnicity as a Covariate (Sensitivity Analysis 1)

eTable 8. Standardized Path Estimates of Parental Factors on Sleep Quality Mediated by Emotion Regulation and Screen Use, Using Multiple Imputation (Sensitivity Analysis 2)

eTable 9. Standardized Path Estimates of Parental Factors on Sleep Timing Mediated by Emotion Regulation and Screen Use, Using Multiple Imputation (Sensitivity Analysis 2)

© 2025 Ge R et al. *JAMA Network Open*.

eTable 10. Standardized Path Estimates of Parental Factors on Sleep Chronotype Mediated by Emotion Regulation and Screen Use, Using Multiple Imputation (Sensitivity Analysis 2)
eTable 11. Moderation Effects of Adolescent Sex: Associations Between Parental Factors and Sleep Outcomes, Using Multiple Imputation (Sensitivity Analysis 2)
eReferences.

This supplementary material has been provided by the authors to give readers additional information about their work.

eMethods. Measures, Mediators, and Statistical Analysis

Measures

Modifiable Parental Factors: Parental factors were both experienced and recorded at ages 9-11 (T1) and were measured via questionnaires during the baseline ABCD assessment.

Parental Warmth. Parental warmth was assessed using the 5-item acceptance subscale of the Child Report of Parent Behavior Inventory (CRPBI),[1-3] as in prior work with the ABCD study dataset.[4] The CRPBI measures youth's perceptions of their caregiver's warmth, acceptance, and responsiveness (e.g., "Believes in showing his/her love for me"). Youth responses were rated on a three-point Likert scale from 1 (Not like him/her) to 3 (A lot like him/her). These items have demonstrated acceptable internal reliability in the ABCD dataset.[5] A total parental acceptance score assessed at T1 was calculated by computing sum scores for the primary caregiver, with higher scores indicating greater levels of parental warmth experienced by the youth.

Parental Monitoring. Parental monitoring was assessed using the 5-item Parental Monitoring Survey (PMS),[6] as in prior studies with the ABCD dataset.[4] The PMS measures youth's perception of their parents' knowledge of their whereabouts and activities (e.g., "How often do your parents/guardians know where you are?"). Youth responses were rated on a five-point Likert scale ranging from 1 (Never) to 5 (Always or almost always). These items have demonstrated moderate internal consistency in the ABCD dataset.[5] A total score at T1 was calculated by summing youth's responses across all five items, with higher scores indicating greater levels of perceived parental monitoring.

Family Conflict. Family conflict was assessed using the 9-item Family Conflict subscale of the Moos Family Environment Scale (FES).[7] The FES measures youth's perceptions of the degree of openly expressed conflict among family members (e.g., "we fight a lot in our family"). Items were scored as 1 (True) or 0 (False). A total score at T1 was calculated by computing sum scores from the youth's responses across all nine items (with appropriate reverse coding), with higher scores indicating greater levels of perceived family conflict.

Parental Psychopathology. Parental psychopathology was assessed using the parent-report 120-item Adult Self Report (ASR), which is part of the Achenbach System of Empirically Based Assessment (ASEBA).[8] The ASR comprises eight syndrome scales measuring different aspects of adult psychopathology, including anxiety/depression, withdrawal, aggression, rule-breaking behaviour, intrusive behaviour, attention problems, thought problems, and other problems. The questionnaire has demonstrated adequate validity and reliability.[9] A total problems score (t-scores) at T1 was computed, with higher scores indicating greater levels of psychopathology experienced by the parent. The ASR can be aggregated into two broad-band scales, internalizing symptoms (i.e., anxious/depressed, withdrawn, and somatic complaints) and externalizing symptoms (i.e., aggressive behaviour, rule-breaking behaviour, and intrusiveness).[10]

Sleep Outcomes

© 2025 Ge R et al. *JAMA Network Open*.

Objective Sleep Measures (Actigraphy). Objective sleep data were collected via actigraphy using Fitbit Charge HR devices worn on participants' non-dominant wrists for three consecutive weeks following their onset visit. Sleep duration, timing and regularity were assessed using the Fitbit devices. Sleep duration and sleep timing were operationalised as the average nightly total sleep time (in minutes) and the average sleep midpoint (in hours from midnight), respectively, across nights. Sleep duration was calculated as the sum of light, deep, and rapid eye movement sleep stages, while sleep timing was calculated as the midpoint between sleep onset (nightly sleep onset in hours from midnight) and sleep offset time (nightly sleep offset in hour from midnight), indicating the deviation from midnight. To calculate mean sleep duration and timing, we followed the methodology of previous studies[11] by computing a weighted average of weekday and weekend recordings. The weighted average was computed as: ((weekday value x weekday day counts) + (weekend value x weekend day counts)) / (weekday day counts + weekend day counts). Sleep regularity was assessed using the Sleep Regularity Index (SRI), a validated metric that quantifies the similarity of sleep patterns across consecutive nights.[107] Following the recommendations of Fischer, Klerman and Phillips [12], for studies exceeding seven days with large sample sizes, SRI should be used to assess sleep regularity. SRI was calculated using the epoch-level sleep/wake data within the R package "GGIR".[13] The specific algorithm used to calculate the SRI is described in detail in the package documentation and related publications.[13,14] The possible range of SRI scores is from 0 (completely random) to 100 (perfect regularity), with higher scores indicating higher sleep regularity. To ensure reliable estimates, only participants with at least seven nights (over the 3-week period) of actigraphy data were included for the computation of sleep duration and timing.[15,16] For sleep regularity, a minimum of six consecutive days was adopted to calculate the SRI. [14]

Polysomnography (PSG) remains the gold standard for sleep assessment, but actigraphy devices demonstrate high agreement (correlations up to 0.97) with PSG for total sleep time and sleep/wake differentiation in healthy populations. [17] A recent validation study shows that while EEG-based devices like Dreem achieve the highest accuracy for sleep staging, consumer trackers such as Fitbit and Oura exhibit moderate agreement with PSG (kappa = 0.45-0.60 for sleep stages) and outperform accelerometry-only devices. [18] Given these findings, Fitbit is considered valid and practical for large-scale, community-based research like ours, where non-invasiveness, multi-night feasibility, and wide deployment in mostly healthy populations are essential.

Sleep Chronotype. Sleep chronotype was assessed using the youth-reported 17-item Munich Chronotype Questionnaire (MCTQ) at T3.[19,20] The MCTQ characterises chronotype by calculating the midpoint between sleep onset and offset on free days over the last four weeks, not dependent on alarm clocks or parental wakeup. This validated measure reliably measures chronotype under natural conditions.[20] The resulting chronotype score, mid-sleep on free days (MSF), reflects the individual's sleep phase preference, with higher values representing a delayed, evening-type chronotype. The MSF ranges from 16 to 40, with each unit corresponding to 1 hour (e.g., 26.33 = 2:20 AM). Full details on the MSF calculation can be found in Kühnle's article.[21]

Sleep Quality. Sleep quality was assessed using the 26-item parent-reported Sleep Disturbance Scale for Children (SDSC)[22] at T3 which evaluates various aspects of youth's general sleep quality and disturbances over the past six months. The SDSC has demonstrated adequate-to-good reliability and validity in community and clinical paediatric samples.[23,24] Items are rated on a 5-point Likert scale ranging from 1 (Never) to 5 (Daily). Total scores range from 26 to 130, with higher scores indicating poorer overall sleep quality.

© 2025 Ge R et al. *JAMA Network Open*.

Mediators

Screen Use. Adolescents' typical screen use was assessed using the Youth Screen Time Survey.[25] Youth reported their usual daily hours spent on six screen types (television shows or movies, videos, video games, texting, video chat, and social media) separately for typical weekdays and weekends. A total daily screen use was calculated as a weighted average across weekdays and weekends: ([weekday hours × 5] + [weekend hours × 2])/7.[26]

Emotion Regulation. Emotion regulation was assessed using the 36-item parent-reported Difficulties in Emotion Regulation Scale (DERS),[27] which measures adolescents' emotion regulation difficulties over a general time frame. The DERS assesses across six domains: difficulties in emotional acceptance, difficulties in goal-directed behaviour, difficulties in impulse control, lack of emotional awareness, lacking emotion regulating strategies, and lacking emotional clarity. Items are rated on a 5-point Likert scale from 1 (Almost never) to 5 (Almost always). A total composite score was calculated by summing the six subscale scores, with higher scores indicating greater difficulties with emotion regulation.

Covariates

Race. Race was included as a categorical variable based on parent-reported baseline demographic surveys. The categories were White, Black, Hispanic, African American, Asian, and other races, following previous work in the ABCD.[28] White was used as the reference profile in all analyses.

Pubertal Status. Pubertal status was measured using the 5-item Pubertal Development Scale (PDS),[29] which assesses physical changes associated with puberty. The PDS is rated on a 4-point Likert scale from 0 (Not yet started) to 4 (Seems completed). Pubertal status was determined based on the total scores of the five items, with higher scores indicating a more advanced pubertal stage. This extensively used measure demonstrates high reliability and validity, with strong correlations to clinician-reported physical exams and hormonal data.[30] Parent-reported PDS data was the primary measure, supplemented by child-report when parent data was missing due to their high inter-rater correlation as well as the availability of data across informants.[31]

Socioeconomic Status(SES). SES was estimated using the Income-to-Needs Ratio (INR), calculated by dividing the reported total household income by the federal poverty threshold for the given household size.[32] The federal poverty thresholds were obtained from the 2017 Federal Register published by the U.S. Department of Health and Human Services. Higher INR scores indicate higher family SES.

We recognise that race and SES are not merely statistical covariates, but rather reflect distinct and intersecting structural dimensions of inequity that shape health opportunities and outcomes. In line with intersectional frameworks, these demographic variables often serve as proxies for lived experience and broader structural conditions.[33] However, our primary research aim was to examine modifiable parental factors, as these are directly actionable within intervention and prevention contexts. SES and race, while important, are not typically modifiable directly by parents and were therefore not the focus of our main analyses. This approach reflects a conceptual distinction between targeting actionable parental behaviours and accounting for broader, less modifiable social determinants. Their intersection may also modify the effects of parenting or other exposures, and future studies would benefit from approaches that explicitly model these intersections rather than treat them solely as individual control variables.

Statistical Analysis

Data Preparation and Handling

To prepare the data for analysis, relevant variables were extracted from the ABCD study, including parental factors at ages 9-10 (T1), mediators at ages 11-12 (T2) and sleep outcomes at ages 13-14 (T3). To minimise the influence of extreme values, all variables were winsorized at or over 3.5 standard deviations. For interpretational clarity, total scores of the SDSC and DERS were reverse-scored so that higher scores indicated better sleep quality and better emotion regulation, respectively.

Normality tests examining skewness and kurtosis indicated no substantial deviation from normality given the large sample size (skew value < |2| or kurtosis < |7| for $n > 300$, per guidelines[34]). Moreover, previous simulation research suggests that linear regression models are robust to moderately skewed distributions in large samples ($n \geq 500$).[35] Therefore, we analysed the original, untransformed data using maximum likelihood estimation with robust standard errors to ensure reliable estimates despite minor departures from normality.

Missing data were primarily addressed using pairwise deletion to maximise the use of available data for each analysis. Participants exceeding 30% missing items on any of the parental factor scales (CRPBI,

© 2025 Ge R et al. *JAMA Network Open*.

PMS, FES, ASR) or the DERS were excluded from analyses involving those measures. For the remaining participants with less than 30% missing data on these scales, imputation using the participant's mean score on completed items for that scale was performed. For linear mixed models, missing data were handled using restricted maximum likelihood estimation, a method well-suited for handling missing data in multilevel models.[36,37] Following recommendations by Sidi and Harel [38], we also conducted sensitivity analyses comparing results obtained through pairwise deletion and multiple imputation to ensure the robustness of our findings to different missing data handling approaches.

Association Between Parental Factors and Sleep Outcomes

Linear mixed models (LMMs) were used to examine the association between parental factors (continuous variable) at T1 and sleep outcomes (continuous) at T3, as recommended by the ABCD study and previous research.[39,40] LMMs accounted for the nested data structure (participants within research sites) by modelling site as a random effect.

Specifically, we used a series of separate LMMs (via the "lme4" package in R) to examine the main effects of each T1 parental factor (warmth, monitoring, family conflict, parental psychopathology) on each T3 sleep outcome (duration, timing, chronotype, regularity, quality) controlling for age at T1, sex, pubertal development and SES. Robust Huber regressions were conducted to address non-normal residuals (Shapiro-Wilk $p < .05$) to provide reliable estimates[41] given the presence of outliers in several predictors and outcome variables. This approach allowed us to determine the contribution of each parental factor to each sleep outcome while controlling for covariates and accounting for the nested data structure.

Standardised beta coefficients (β) and corresponding 95% confidence intervals (CIs) were computed to indicate the predictive strength of parental factors on sleep outcomes after accounting for covariates. To correct for multiple comparisons across the 20 tests (four parental factors X five sleep outcomes), Benjamini & Hochberg false discovery rate (FDR) corrections were applied[42] to balance the risks of type I and type II errors.

Mediation Analysis

For significant associations between parental factors and adolescent sleep outcomes, mediation analyses were conducted using the PROCESS v4.5 Model 4 macro in R.[43]This approach allowed us to examine the potential indirect effects of two T2 mediators – screen use and emotion regulation – on the relationship between T1 parental factors and T3 sleep outcomes. We controlled for the same covariates as in the main effects analyses and included site as a random effect to account for the nested data structure.

Indirect paths were estimated via bootstrapping approach (with 1,000 iterations). This method is preferred due to its reduced bias compared to traditional methods,[44,45] and its robustness to violations of normality.[46] Bias-corrected bootstrap CIs were generated for the indirect effects, with a 95% CI excluding zero indicating a statistically significant indirect effect.[47]

The proportion mediated (PM) was calculated as the ratio of the indirect effect (IE; also known as the natural indirect effect, NIE) to the total effect (TE), representing the proportion of the total effect that operates through the mediator.[48] It is important to note that PM values can be unstable or potentially misleading when the total effect is small or nonsignificant, as even small indirect effects can result in disproportionately large PM values. PM should not be interpreted as the expected change in the outcome following an intervention on the mediator; this interpretation aligns more closely with alternative metrics such as the proportion eliminated.[49] We encourage readers to consider both the absolute size of the total effect and the PM when interpreting mediation results.

Moderation Analysis

To explore potential sex differences, we conducted exploratory moderation analyses by adding interaction terms between sex and each parental factor (e.g., parental warmth * sex) to the LMMs. These analyses involved calculating the slopes of the association between parental factors and sleep outcomes at different levels of the sex moderator (female, male). All models controlled for covariates and included site as a random effect. To account for multiple comparisons, Benjamini & Hochberg's FDR correction was applied for multiple comparisons.[42]

For models exhibiting significant sex interaction effects, simple slopes analysis ("emmeans" package in R) was conducted. This method allowed for the examination of the relationship between each parental factor and the sleep outcome at different levels (male or female) of the moderating variable (sex).

© 2025 Ge R et al. *JAMA Network Open*.

This approach allowed for conditional effects of parental factors on sleep outcomes depending on the sex of the adolescent.[50,51] In cases of significant moderation by sex, we then investigated the effects of the mediators separately for males and females.

Sensitivity Analysis

Two sensitivity analyses were conducted to evaluate the robustness of the findings and the potential impact of methodological decisions on the results.

Given the strong association between race and SES[52], we did not include race as a covariate in our main analyses. However, to examine the possible confounding effect of race on our primary analyses, we conducted sensitivity analyses with race included as an additional covariate, using the White ethnic group as the reference category. It is important to note that our interest was not in the main effect of race on sleep outcomes but rather in the effect of parenting while additionally controlling for race.

We compared the results derived from pairwise deletion with those obtained using multiple imputation (MI). By generating multiple plausible datasets with imputed values for the missing data, MI could account for uncertainty associated with missingness.[53,54] We performed MI on the full sample of 3,419 participants using the "mice" package[55] in R. Predictive mean matching (PMM), a method well-suited for non-normal data,[56,57] was used for imputing continuous variables (e.g., sleep regularity), while logistic regression was used for categorical variables (e.g., sex), following recommendations for handling different data types.[55] By comparing findings from both approaches, we could assess whether our results were sensitive to the chosen missing data handling method.

© 2025 Ge R et al. *JAMA Network Open*.

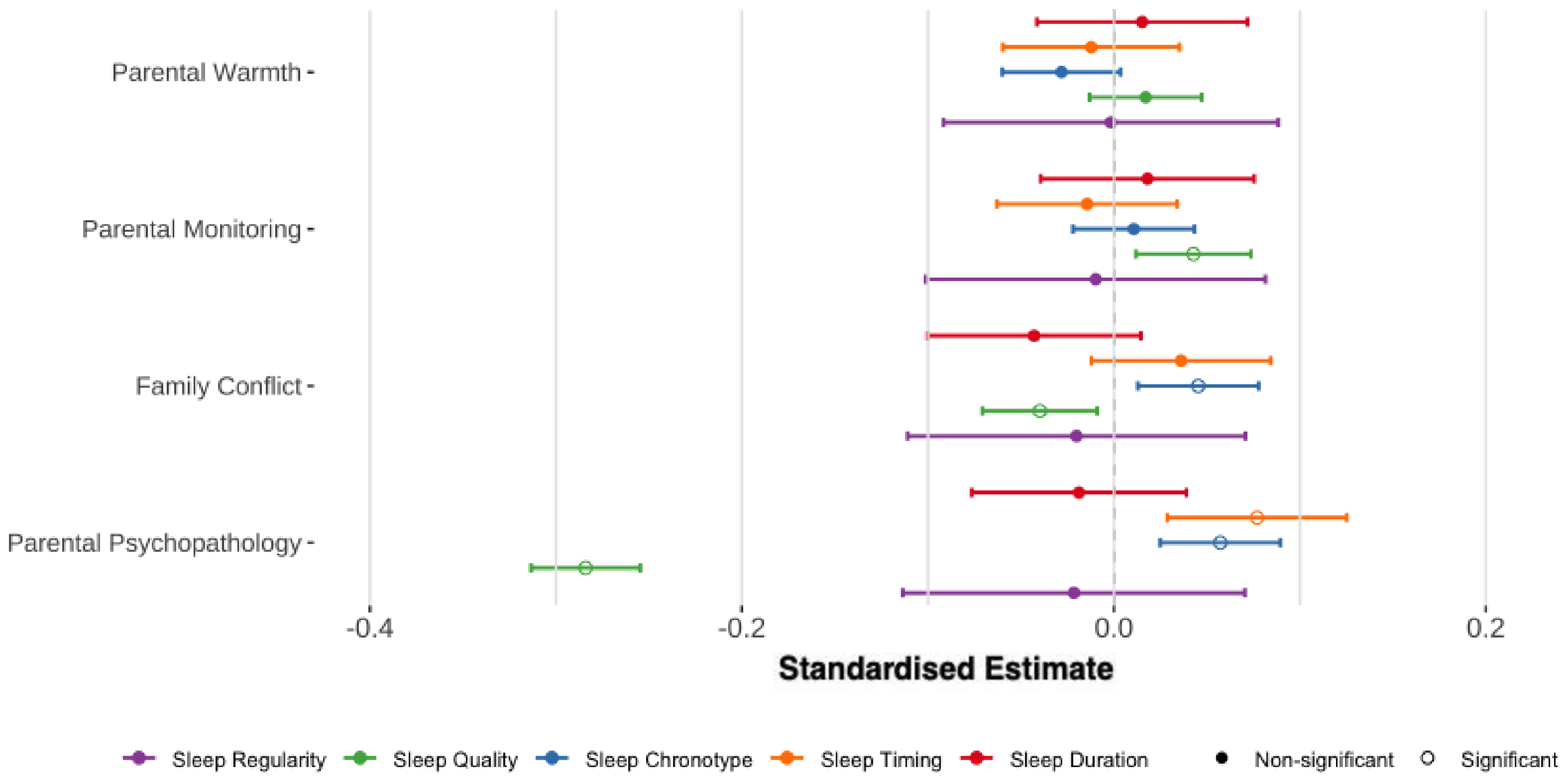

eFigure 1. Associations Between Parental Factors and Sleep Outcomes, Adjusted for Race (Sensitivity Analysis 1)

Standardised parameter estimates and 95% confidence intervals from linear mixed models depicting the relationships between parental factors (parental warmth, parental monitoring, family conflict, and parental psychopathology) measured at baseline and five sleep outcomes (sleep duration, sleep timing, sleep chronotype, sleep quality, and sleep regularity) measured four years later. All models were controlled for age, sex, pubertal status, race, income-to-needs ratio. Site was modelled as a random effect. The dashed vertical line represents the null effect (estimate = 0).

© 2025 Ge R et al. *JAMA Network Open*.

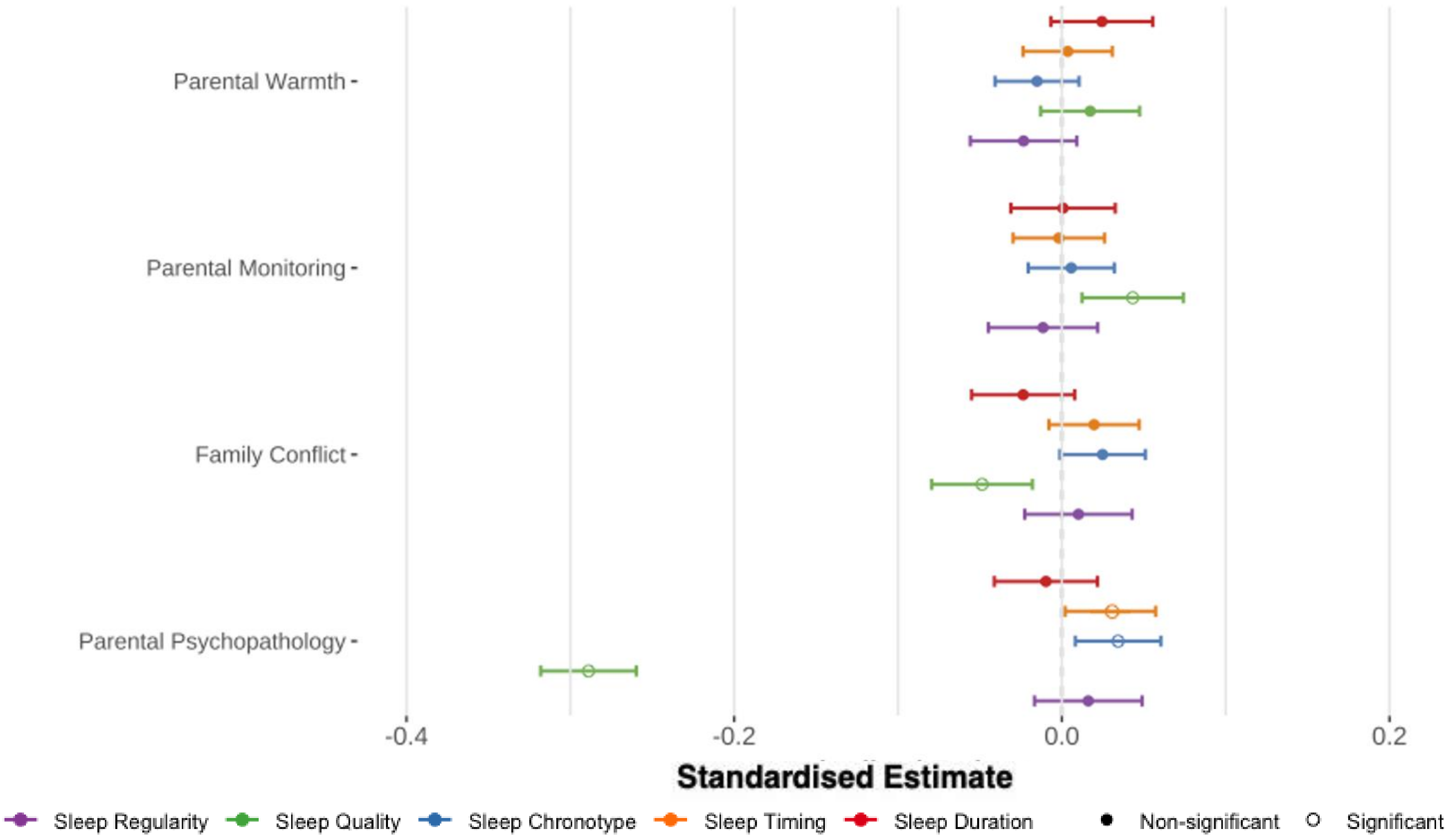

eFigure 2. Associations Between Parental Factors and Sleep Outcomes Using Imputation (Sensitivity Analysis 2)

Standardised parameter estimates and 95% confidence intervals from linear mixed models depicting the relationships between parental factors (parental warmth, parental monitoring, family conflict, and parental psychopathology) measured at baseline and five sleep outcomes (sleep duration, sleep timing, sleep chronotype, sleep quality, and sleep regularity) measured four years later. The analysis employed multiple imputation to address missing data. All models were controlled for age, sex, pubertal status, income-to-needs ratio. Site was modelled as a random effect. The dashed vertical line represents the null effect (estimate = 0).

© 2025 Ge R et al. *JAMA Network Open*.

eTable 1. Comparison of Included vs Excluded Participants on Key Demographics at Baseline

Characteristic	Overall (N=11,868) Mean (SD)	Included (N=3,419) Mean (SD)	Excluded (N =8,449) Mean (SD)
Age, y, No. (%)	9.48 (0.51)	9.49 (0.50)	9.47 (0.51)
Biological sex			
Female	5,677 (47.8%)	1,642 (48.0%)	3,509 (48.5%)
Male	6,188 (52.1%)	1,828 (52.0%)	3,724 (51.5%)
Race and ethnicity[a], No. (%)			
Asian	252 (2.1%)	187 (2.5%)	129 (1.8%)
Black	1,784 (15.0%)	332 (9.6%)	1,107 (15.3%)
Hispanic	2,410 (20.3%)	698 (20.0%)	1,340 (18.5%)
White	6,173 (52.0%)	2,009 (58.0%)	3,874 (53.6%)
Other race	1,247 (10.5%)	344 (9.9%)	782 (10.8%)
Pubertal status[b]	1.75 (0.87)	1.75 (0.85)	
Income-to-needs ratio[c]	3.94 (2.86)	4.17 (2.88)	3.83 (2.85)

[a] Race and ethnicity data were collected by the parent report.
[b] Puberty status was measured using the Pubertal Development Scale, with higher scores indicating a more advanced pubertal stage.
[c] Income to needs ratio was calculated following established methods by Gonzalez, et al.[32]

© 2025 Ge R et al. *JAMA Network Open*.

eTable 2. Site-Specific Descriptive Statistics for the ABCD Cohort at Baseline

**Variable**	**N**	**site01** N = 93[1]	**site02** N = 168[1]	**site03** N = 229[1]	**site04** N = 327[1]	**site05** N = 113[1]	**site06** N = 245[1]	**site07** N = 17[1]	**site08** N = 99[1]	**site09** N = 89[1]	**site10** N = 268[1]	**site11** N = 49[1]	**site12** N = 165[1]	**site13** N = 255[1]	**site14** N = 89[1]	**site15** N = 103[1]	**site16** N = 302[1]	**site17** N = 126[1]	**site18** N = 137[1]	**site19** N = 131[1]	**site20** N = 156[1]	**site21** N = 232[1]	**site22** N = 26[1]
**Sex**	3,419																						
female		44 (47%)	74 (44%)	104 (45%)	159 (49%)	50 (44%)	125 (51%)	5 (29%)	53 (54%)	38 (43%)	125 (47%)	25 (51%)	88 (53%)	125 (49%)	38 (43%)	47 (46%)	134 (44%)	53 (42%)	63 (46%)	70 (53%)	77 (49%)	102 (44%)	12 (46%)
male		49 (53%)	94 (56%)	125 (55%)	168 (51%)	63 (56%)	120 (49%)	12 (71%)	46 (46%)	51 (57%)	143 (53%)	24 (49%)	77 (47%)	130 (51%)	51 (57%)	56 (54%)	168 (56%)	73 (58%)	74 (54%)	61 (47%)	79 (51%)	130 (56%)	14 (54%)
**Age**	3,419	9.43 (0.52)	9.60 (0.49)	9.47 (0.50)	9.42 (0.51)	9.38 (0.49)	9.58 (0.49)	9.24 (0.44)	9.58 (0.50)	9.47 (0.50)	9.44 (0.50)	9.57 (0.50)	9.44 (0.50)	9.39 (0.49)	9.78 (0.42)	9.49 (0.50)	9.48 (0.51)	9.44 (0.50)	9.51 (0.50)	9.56 (0.51)	9.63 (0.48)	9.47 (0.50)	9.62 (0.50)
**Race**	3,419																						
Black		4 (4.3%)	0 (0%)	21 (9.2%)	34 (10%)	13 (12%)	2 (0.8%)	1 (5.9%)	2 (2.0%)	9 (10%)	14 (5.2%)	8 (16%)	33 (20%)	29 (11%)	5 (5.6%)	46 (45%)	1 (0.3%)	0 (0%)	7 (5.1%)	31 (24%)	42 (27%)	23 (9.9%)	1 (3.8%)
Asian		6 (6.5%)	2 (1.2%)	3 (1.3%)	3 (0.9%)	1 (0.9%)	6 (2.4%)	0 (0%)	19 (19%)	6 (6.7%)	7 (2.6%)	0 (0%)	6 (3.6%)	9 (3.5%)	1 (1.1%)	0 (0%)	0 (0%)	5 (4.0%)	4 (2.9%)	1 (0.8%)	0 (0%)	4 (1.7%)	2 (7.7%)
Hispanic		43 (46%)	30 (18%)	179 (78%)	58 (18%)	3 (2.7%)	22 (9.0%)	3 (18%)	11 (11%)	22 (25%)	160 (60%)	4 (8.2%)	12 (7.3%)	23 (9.0%)	4 (4.5%)	1 (1.0%)	22 (7.3%)	5 (4.0%)	9 (6.6%)	9 (6.9%)	6 (3.8%)	47 (20%)	4 (15%)
Other		14 (15%)	13 (7.7%)	6 (2.6%)	73 (22%)	9 (8.0%)	31 (13%)	2 (12%)	20 (20%)	13 (15%)	20 (7.5%)	4 (8.2%)	27 (16%)	32 (13%)	3 (3.4%)	12 (12%)	14 (4.6%)	5 (4.0%)	7 (5.1%)	9 (6.9%)	11 (7.1%)	13 (5.6%)	2 (7.7%)
White		26 (28%)	123 (73%)	20 (8.7%)	159 (49%)	87 (77%)	184 (75%)	11 (65%)	47 (47%)	39 (44%)	67 (25%)	33 (67%)	87 (53%)	162 (64%)	76 (85%)	44 (43%)	265 (88%)	111 (88%)	110 (80%)	81 (62%)	97 (62%)	145 (63%)	17 (65%)
**INR**	3,419	4.47 (3.63)	4.99 (2.68)	2.41 (2.15)	3.07 (2.40)	4.76 (2.79)	5.11 (2.70)	3.96 (2.99)	7.05 (3.27)	6.24 (3.39)	3.12 (2.92)	3.42 (2.72)	5.11 (2.99)	4.31 (2.70)	4.85 (2.34)	2.48 (2.26)	3.48 (2.10)	5.24 (2.58)	4.97 (2.46)	4.04 (2.57)	3.28 (2.39)	4.82 (2.95)	6.98 (3.21)

[1]n (%); Mean (SD)

eTable 3. Moderation Role of Adolescent Sex on the Associations Between Parental Factors and Sleep Outcomes

	**Parental Warmth × Sex**		**Parental Monitoring × Sex**		**Family Conflict × Sex**		**Parental Psychopathology × Sex**	
**Sleep Outcomes**	*Estimates*	*CI*	*Estimates*	*CI*	*Estimates*	*CI*	*Estimates*	*CI*
Sleep Duration	-0.65	-5.31 – 3.98	2.25	-2.61 – 7.16	0.84	-3.74 – 5.45	1.84	-2.96 – 6.64
Sleep Timing	-0.002	-0.16 – 0.15	0.04	-0.15 – 0.23	0.09	-0.09 – 0.27	-0.01	-0.20 – 0.18
Sleep Chronotype	**0.16****	0.04 – 0.27	5.318e-03	-0.12 – 0.13	0.03	-0.09 – 0.16	0.10	-0.02 – 0.22
Sleep Quality	-0.11	-0.44 – 0.22	0.09	-0.25 – 0.42	0.03	-0.30 – 0.36	-0.21	-0.52 – 0.09
Sleep Regularity	3.57	-0.07 – 6.37	3.48	-0.50 – 6.46	-0.74	-3.52 – 2.05	1.21	-1.79 – 4.21

Note: Estimates represent the unstandardised beta coefficients for the interaction terms between each parental factor and sex (male) in separate regression models, controlling for main effects of the parental factor, sex, and covariates. CI represents the 95% confidence interval for the interaction effect. Values in bold indicate statistical significance after false-discovery rate correction. ** $p < .01$

eTable 4. Standardized Path Estimates of Parental Factors on Sleep Quality Mediated by Emotion Regulation and Screen Use, Including Race and Ethnicity as a Covariate (Sensitivity Analysis 1)

**Direct Effects**	**Estimate, 95% CI**
Parental Monitoring	**0.31** (0.04, 0.58)
Family Conflict	-0.25 (-0.50, 0.01)
Parental Psychopathology	**-1.90** (-2.16, -1.64)
**Indirect Effects**	**Estimate, 95% CI**
Parental Monitoring	
via Emotion Regulation	0.07 (-0.002, 0.143)
via Screen Use	**0.03** (0.01, 0.06)
Family Conflict	
via Emotion Regulation	**-0.09** (-0.16, -0.03)
via Screen Use	**-0.04** (-0.07, -0.02)
Parental Psychopathology	
via Emotion Regulation	**-0.04** (-0.43, -0.28)
via Screen Use	**-0.02** (-0.05, -0.01)

Note: Sensitivity analysis was conducted by including race as a covariate in the analysis. Unstandardised coefficients and 95% confidence intervals (CI) are presented. Bold values indicate statistically significant associations within this table.

eTable 5. Standardised Path Estimates of Parental Factors on Sleep Timing Mediated by Emotion Regulation and Screen Use, Including Race and Ethnicity as a Covariate (Sensitivity Analysis 1)

**Direct Effects**	**Estimate, 95% CI**
Parental Psychopathology	**0.12** (0.03, 0.22)
**Indirect Effects**	**Estimate, 95% CI***
Parental Psychopathology	
via Emotion Regulation	-0.017 (-0.037, 0.002)
via Screen Use	0.004 (-0.005, 0.014)

Note: Sensitivity analysis was conducted by including race as a covariate in the analysis. Unstandardised coefficients and 95% confidence intervals (CI) are presented. Bold values indicate statistically significant associations within this table.

eTable 6. Standardized Path Estimates of Parental Factors on Sleep Chronotype Mediated by Emotion Regulation and Screen Use, Including Race and Ethnicity as a Covariate (Sensitivity Analysis 1)

**Direct Effects**	**Estimate, 95% CI**
Family Conflict	**0.07** (0.02, 0.13)
Parental Psychopathology	**0.08** (0.01, 0.14)
**Indirect Effects**	**Estimate, 95% CI***
Family Conflict	
via Emotion Regulation	-0.001 (-0.004, 0.002)
via Screen Use	**0.02** (0.01, 0.03)
Parental Psychopathology	
via Emotion Regulation	-0.01 (-0.02, 0.01)
via Screen Use	**0.02** (0.01, 0.03)

Note: Sensitivity analysis was conducted by including race as a covariate in the analysis. Unstandardised coefficients and 95% confidence intervals (CI) are presented. Bold values indicate statistically significant associations within this table.

eTable 7. Moderation Effects of Adolescent Sex: Associations Between Parental Factors and Sleep Outcomes, Including Race and Ethnicity as a Covariate (Sensitivity Analysis 1)

	**Parental Warmth × Sex**		**Parental Monitoring × Sex**		**Family Conflict × Sex**		**Parental Psychopathology × Sex**	
**Sleep Outcomes**	*Estimates*	*CI*	*Estimates*	*CI*	*Estimates*	*CI*	*Estimates*	*CI*
Sleep Duration	-0.98	-5.53 – 3.57	0.30	-4.48 – 5.12	2.56	-1.98 – 7.12	2.16	-2.71 – 7.01
Sleep Timing	1.09	-0.08 – 0.27	0.14	-0.04 – 0.33	0.05	-0.13 – 0.22	0.05	-0.14 – 0.23
Sleep Chronotype	**0.16****	0.05 – 0.27	1.23	-0.04 – 0.19	-0.01	-0.12 – 0.10	0.06	-0.06 – 0.17
Sleep Quality	-0.18	-0.32 – 0.68	0.24	-0.27 – 0.76	-0.23	-0.73 – 0.27	0.13	-0.36 – 0.62
Sleep Regularity	2.06	-0.67 – 4.78	3.23^	0.35 – 6.12	-0.17	0.35 – 2.59	1.65	-1.31 – 4.62

Note: Sensitivity analysis was conducted by including race/ethnicity as a covariate in the analysis. Estimates represent the unstandardised beta coefficients for the interaction terms between each parental factor and sex (male) in separate regression models, controlling for main effects of the parental factor, sex, and covariates for age, race/ethnicity, income-to-needs ratio. CI represents the 95% confidence interval for the interaction effect. Values in bold survive correction using the False Discovery Rate (FDR), alpha = 0.05, corrected across all predictors within the table. ** $p < .01$. ^ The association did not survive FDR correction for multiple comparisons.

eTable 8. Standardized Path Estimates of Parental Factors on Sleep Quality Mediated by Emotion Regulation and Screen Use, Using Multiple Imputation (Sensitivity Analysis 2)

**Direct Effects**	**Estimate, 95% CI**
Parental Monitoring	**0.32** (0.07, 0.58)
Family Conflict	**-0.28** (-0.06, -0.03)
Parental Psychopathology	**-1.92** (-2.17, -1.67)
**Indirect Effects**	**Estimate, 95% CI**
Parental Monitoring	
via Emotion Regulation	0.06 (-0.01, 0.13)
via Screen Use	**0.04** (0.02, 0.07)
Family Conflict	
via Emotion Regulation	**-0.11** (-0.18, -0.04)
via Screen Use	**-0.04** (-0.07, -0.02)
Parental Psychopathology	
via Emotion Regulation	**-0.39** (-0.47, -0.31)
via Screen Use	**-0.03** (-0.05, -0.01)

Note: Sensitivity analysis was conducted by using multiple imputation. Unstandardised coefficients and 95% confidence intervals (CI) are presented. Bold values indicate statistically significant associations within this table.

eTable 9. Standardized Path Estimates of Parental Factors on Sleep Timing Mediated by Emotion Regulation and Screen Use, Using Multiple Imputation (Sensitivity Analysis 2)

**Direct Effects**	**Estimate, 95% CI**
Parental Psychopathology	0.05 (-0.01, 0.09)
**Indirect Effects**	**Estimate, 95% CI***
Parental Psychopathology	
via Emotion Regulation	0.004 (-0.008, 0.016)
via Screen Use	<0.0001 (-0.003, 0.004)

Note: Sensitivity analysis was conducted by using multiple imputation. Unstandardised coefficients and 95% confidence intervals (CI) are presented. Bold values indicate statistically significant associations within this table.

eTable 10. Standardized Path Estimates of Parental Factors on Sleep Chronotype Mediated by Emotion Regulation and Screen Use, Using Multiple Imputation (Sensitivity Analysis 2)

**Direct Effects**	**Estimate, 95% CI**
Family Conflict	**0.050** (0.003, 0.096)
Parental Psychopathology	0.05 (0.02, 0.07)
**Indirect Effects**	**Estimate, 95% CI***
Family Conflict	
via Emotion Regulation	<0.001 (-0.002, 0.003)
via Screen Use	**0.01** (0.01, 0.02)
Parental Psychopathology	
via Emotion Regulation	<-0.001 (-0.01, 0.01)
via Screen Use	**0.007** (0.003, 0.094)

Note: Sensitivity analysis was conducted by using multiple imputation. Unstandardised coefficients and 95% confidence intervals (CI) are presented. Bold values indicate statistically significant associations within this table.

eTable 11. Moderation Effects of Adolescent Sex: Associations Between Parental Factors and Sleep Outcomes, Using Multiple Imputation (Sensitivity Analysis 2)

	**Parental Warmth × Sex**		**Parental Monitoring × Sex**		**Family Conflict × Sex**		**Parental Psychopathology × Sex**	
**Sleep Outcomes**	*Estimates*	*CI*	*Estimates*	*CI*	*Estimates*	*CI*	*Estimates*	*CI*
Sleep Duration	0.01	-2.13 – 2.16	0.13	-2.11 – 2.38	1.27	-0.89 – 3.42	1.61	-0.54 – 3.77
Sleep Timing	0.01	-0.09 – 0.10	0.04	-0.05 – 0.14	0.01	-0.08 – 0.10	-0.01	-0.10 – 0.08
Sleep Chronotype	0.07	-0.01 – 0.16	0.0006	-0.09 – 0.09	0.009	-0.08 – 0.09	0.06	-0.03 – 0.14
Sleep Quality	0.16	-0.35 – 0.66	0.27	-0.24 – 0.79	-0.14	-0.65 – 0.37	0.15	-0.34 – 0.64
Sleep Regularity	0.71	-0.12 – 1.55	0.81	-0.06 – 1.69	-0.25	-1.08 – 0.58	0.35	-0.48 – 1.19

Note: Sensitivity analysis was conducted by using multiple imputation. Estimates represent the unstandardised beta coefficients for the interaction terms between each parental factor and sex (male) in separate regression models, controlling for main effects of the parental factor, sex, and covariates. CI represents the 95% confidence interval for the interaction effect. Values in bold survive correction using the False Discovery Rate (FDR), alpha = 0.05, corrected across all predictors within the table.

eReferences.

1. Schaeffer P, Asnes AG. What Do Pediatricians Tell Parents About Bed-Sharing? *Matern Child Health J*. Jan 2018;22(1):51-58. doi:10.1007/s10995-017-2353-5

2. Schaefer ES. Children's Reports of Parental Behavior: An Inventory. *Child Dev*. Jun 1965;36(2):413-24. doi:10.2307/1126465

3. Schludermann S, Schludermann E. Questionnaire for children and youth (CRPBI-30). *Unpublished manuscript, University of Manitoba, Winnipeg*. 1988;

4. DeVille DC, Whalen D, Breslin FJ, et al. Prevalence and Family-Related Factors Associated With Suicidal Ideation, Suicide Attempts, and Self-injury in Children Aged 9 to 10 Years. *JAMA Netw Open*. Feb 5 2020;3(2):e1920956. doi:10.1001/jamanetworkopen.2019.20956

5. Gonzalez R, Thompson EL, Sanchez M, et al. An update on the assessment of culture and environment in the ABCD Study®: Emerging literature and protocol updates over three measurement waves. *Dev Cogn Neurosci*. Dec 2021;52:101021. doi:10.1016/j.dcn.2021.101021

6. Karoly HC, Callahan T, Schmiege SJ, Ewing SW. Evaluating the Hispanic Paradox in the Context of Adolescent Risky Sexual Behavior: The Role of Parent Monitoring. *J Pediatr Psychol*. May 2016;41(4):429-40. doi:10.1093/jpepsy/jsv039

7. Moos RH, Moos BS. A typology of family social environments. *Fam Process*. Dec 1976;15(4):357-71. doi:10.1111/j.1545-5300.1976.00357.x

8. Achenbach TM, Verhulst F. Achenbach system of empirically based assessment (ASEBA). *Burlington, Vermont*. 2010;

9. Achenbach TM. *Manual for the young adult self-report and young adult behavior checklist*. University of Vermont, Department of Psychiatry; 1997.

10. Guerrero M, Hoffmann M, Pulkki-Råback L. Psychometric Properties of the Adult Self-Report: Data from over 11,000 American Adults. *Stats*. Dec 2020;3(4):465-474. doi:10.3390/stats3040029

11. Lauderdale DS, Knutson KL, Yan LL, Liu K, Rathouz PJ. Self-reported and measured sleep duration: how similar are they? *Epidemiology*. Nov 2008;19(6):838-45. doi:10.1097/EDE.0b013e318187a7b0

12. Fischer D, Klerman EB, Phillips AJK. Measuring sleep regularity: theoretical properties and practical usage of existing metrics. *Sleep*. Oct 11 2021;44(10)doi:10.1093/sleep/zsab103

13. Windred DP, Jones SE, Russell A, et al. Objective assessment of sleep regularity in 60 000 UK Biobank participants using an open-source package. *Sleep*. Dec 10 2021;44(12)doi:10.1093/sleep/zsab254

14. Windred DP, Burns AC, Lane JM, et al. Sleep regularity is a stronger predictor of mortality risk than sleep duration: A prospective cohort study. *Sleep*. Jan 11 2024;47(1)doi:10.1093/sleep/zsad253

15. Aili K, Astrom-Paulsson S, Stoetzer U, Svartengren M, Hillert L. Reliability of Actigraphy and Subjective Sleep Measurements in Adults: The Design of Sleep Assessments. *J Clin Sleep Med*. Jan 15 2017;13(1):39-47. doi:10.5664/jcsm.6384

16. Sadeh A. Iii. Sleep assessment methods. *Monogr Soc Res Child Dev*. Mar 2015;80(1):33-48. doi:10.1111/mono.12143

17. Ancoli-Israel S, Cole R, Alessi C, Chambers M, Moorcroft W, Pollak CP. The Role of Actigraphy in the Study of Sleep and Circadian Rhythms. *Sleep*. 2003;26(3):342-392. doi:10.1093/sleep/26.3.342

18. Ong JL, Golkashani HA, Ghorbani S, et al. Selecting a sleep tracker from EEG-based, iteratively improved, low-cost multisensor, and actigraphy-only devices. *Sleep Health*. Feb 2024;10(1):9-23. doi:10.1016/j.sleh.2023.11.005

19. Roenneberg T, Keller LK, Fischer D, Matera JL, Vetter C, Winnebeck EC. Human activity and rest in situ. *Methods Enzymol*. 2015;552:257-83. doi:10.1016/bs.mie.2014.11.028

20. Zavada A, Gordijn MC, Beersma DG, Daan S, Roenneberg T. Comparison of the Munich Chronotype Questionnaire with the Horne-Ostberg's Morningness-Eveningness Score. *Chronobiol Int*. 2005;22(2):267-78. doi:10.1081/cbi-200053536

21. Kühnle T. *Quantitative analysis of human chronotypes*. lmu; 2006.

22. Bruni O, Ottaviano S, Guidetti V, et al. The Sleep Disturbance Scale for Children (SDSC). Construction and validation of an instrument to evaluate sleep disturbances in childhood and adolescence. *J Sleep Res*. Dec 1996;5(4):251-61. doi:10.1111/j.1365-2869.1996.00251.x

23. Ferreira VR, Carvalho LB, Ruotolo F, de Morais JF, Prado LB, Prado GF. Sleep disturbance scale for children: translation, cultural adaptation, and validation. *Sleep Med*. 2009;10(4):457-463.

24. Bruni O, Ottaviano S, Guidetti V, et al. The Sleep Disturbance Scale for Children (SDSC) Construct ion and validation of an instrument to evaluate sleep disturbances in childhood and adolescence. *J Sleep Res*. 1996;5(4):251-261.

25. Hisler GC, Hasler BP, Franzen PL, Clark DB, Twenge JM. Screen media use and sleep disturbance symptom severity in children. *Sleep Health*. Dec 2020;6(6):731-742. doi:10.1016/j.sleh.2020.07.002

26. Nagata JM, Singh G, Yang JH, et al. Bedtime screen use behaviors and sleep outcomes: Findings from the Adolescent Brain Cognitive Development (ABCD) Study. *Sleep Health*. Aug 2023;9(4):497-502. doi:10.1016/j.sleh.2023.02.005

27. Gratz KL, Roemer L. Multidimensional assessment of emotion regulation and dysregulation: Development, factor structure, and initial validation of the difficulties in emotion regulation scale. *Journal of Psychopathology and Behavioral Assessment*. 2004;26(1):41-54. doi:10.1023/B:JOBA.0000007455.08539.94

28. Isaiah A, Ernst T, Cloak CC, Clark DB, Chang L. Associations between frontal lobe structure, parent-reported obstructive sleep disordered breathing and childhood behavior in the ABCD dataset. *Nat Commun*. Apr 13 2021;12(1):2205. doi:10.1038/s41467-021-22534-0

29. Petersen AC, Crockett L, Richards M, Boxer A. A self-report measure of pubertal status: Reliability, validity, and initial norms. *J Youth Adolesc*. Apr 1988;17(2):117-33. doi:10.1007/BF01537962

30. Shirtcliff EA, Dahl RE, Pollak SD. Pubertal development: correspondence between hormonal and physical development. *Child Dev*. Mar-Apr 2009;80(2):327-37. doi:10.1111/j.1467-8624.2009.01263.x

31. Thijssen S, Collins PF, Luciana M. Pubertal development mediates the association between family environment and brain structure and function in childhood. *Dev Psychopathol*. May 2020;32(2):687-702. doi:10.1017/S0954579419000580

32. Gonzalez MR, Palmer CE, Uban KA, Jernigan TL, Thompson WK, Sowell ER. Positive Economic, Psychosocial, and Physiological Ecologies Predict Brain Structure and Cognitive Performance in 9-10-Year-Old Children. Original Research.

Front Hum Neurosci. 2020-October-28 2020;14:578822. doi:10.3389/fnhum.2020.578822

33. Simkus A, Holtz KD, Twombly EC. An intersectionality framework for identifying relevant covariates in health equity research. Review. *Front*. 2024-March-14 2024;Volume 12 - 2024doi:10.3389/fpubh.2024.1286121

34. Kim HY. Statistical notes for clinical researchers: assessing normal distribution (2) using skewness and kurtosis. *Restor Dent Endod*. Feb 2013;38(1):52-4. doi:10.5395/rde.2013.38.1.52

35. Lumley T, Diehr P, Emerson S, Chen L. The importance of the normality assumption in large public health data sets. *Annu Rev Public Health*. 2002;23:151-69. doi:10.1146/annurev.publhealth.23.100901.140546

36. West BT, Welch KB, Galecki AT. *Linear mixed models: a practical guide using statistical software*. Chapman and Hall/CRC; 2022.

37. Nagin DS, Odgers CL. Group-based trajectory modeling in clinical research. *Annu Rev Clin Psychol*. 2010;6:109-38. doi:10.1146/annurev.clinpsy.121208.131413

38. Sidi Y, Harel O. The treatment of incomplete data: Reporting, analysis, reproducibility, and replicability. *Soc Sci Med*. Jul 2018;209:169-173. doi:10.1016/j.socscimed.2018.05.037

39. Paulus MP, Squeglia LM, Bagot K, et al. Screen media activity and brain structure in youth: Evidence for diverse structural correlation networks from the ABCD study. *Neuroimage*. 2019;185:140-153.

40. Dick A, Garcia N, Pruden S, et al. No evidence for a bilingual executive function advantage in the nationally representative ABCD study. Nature Human Behaviour, 3 (7), 692-701. 2019.

41. Huber PJ. *Robust statistics*. vol 523. John Wiley & Sons; 2004.

42. Benjamini Y, Hochberg Y. Controlling the False Discovery Rate - a Practical and Powerful Approach to Multiple Testing. *Journal of the Royal Statistical Society Series B-Statistical Methodology*. 1995;57(1):289-300. doi:DOI 10.1111/j.2517-6161.1995.tb02031.x

43. Hayes AF. *Introduction to mediation, moderation, and conditional process analysis: A regression-based approach*. Guilford publications; 2017.

44. Shrout PE, Bolger N. Mediation in experimental and nonexperimental studies: new procedures and recommendations. *Psychological methods*. 2002;7(4):422.

45. MacKinnon DP, Lockwood CM, Williams J. Confidence limits for the indirect effect: Distribution of the product and resampling methods. *Multivariate behavioral research*. 2004;39(1):99-128.

46. Hayes AF, Scharkow M. The relative trustworthiness of inferential tests of the indirect effect in statistical mediation analysis: does method really matter? *Psychological science*. 2013;24(10):1918-1927.

47. Mackinnon DP, Lockwood CM, Williams J. Confidence Limits for the Indirect Effect: Distribution of the Product and Resampling Methods. *Multivariate Behav Res*. Jan 1 2004;39(1):99. doi:10.1207/s15327906mbr3901_4

48. VanderWeele TJ. Policy-Relevant Proportions for Direct Effects. *Epidemiology*. 2013;24(1)

49. Suzuki E, Mitsuhashi T, Tsuda T, Yamamoto E. Alternative Definitions of "Proportion Eliminated". *Epidemiology*. 2014;25(2):308-309. doi:10.1097/ede.0000000000000050

50. Aiken LS, West SG, Reno RR. *Multiple regression: Testing and interpreting interactions*. sage; 1991.

51. Preacher KJ, Curran PJ, Bauer DJ. Computational tools for probing interactions in multiple linear regression, multilevel modeling, and latent curve analysis. *Journal of educational and behavioral statistics*. 2006;31(4):437-448.

52. Cheng TL, Goodman E, RESEARCH TCOP, et al. Race, Ethnicity, and Socioeconomic Status in Research on Child Health. *Pediatrics*. 2015;135(1):e225-e237. doi:10.1542/peds.2014-3109

53. Schafer JL, Graham JW. Missing data: our view of the state of the art. *Psychological methods*. 2002;7(2):147.

54. Rubin RB, McHugh MP. Development of parasocial interaction relationships. 1987;

55. Van Buuren S, Groothuis-Oudshoorn K. mice: Multivariate imputation by chained equations in R. *Journal of statistical software*. 2011;45:1-67.

56. Rubin DB, Schenker N. Multiple imputation for interval estimation from simple random samples with ignorable nonresponse. *Journal of the American statistical Association*. 1986;81(394):366-374.

57. Little RJ. A test of missing completely at random for multivariate data with missing values. *Journal of the American statistical Association*. 1988;83(404):1198-1202.
