# Supplementary material for: Modifiable Parental Factors and Adolescent Sleep During Early Adolescence
Source: JAMA Netw Open. 2025 Sep 11;8(9):e2531333. doi: 10.1001/jamanetworkopen.2025.31333 (PMC12426799; doi:10.1001/jamanetworkopen.2025.31333)
Supplement: Supplement 2. — Data Sharing Statement [file jamanetwopen-e2531333-s002.pdf]

Data Sharing Statement

Ge. Modifiable Parental Factors and Adolescent Sleep During Early Adolescence. *JAMA Netw Open*. Published September 11, 2025. doi:10.1001/jamanetworkopen.2025.31333

Data
Data available: No

Additional Information
Explanation for why data not available: Only researchers with an approved NDA Data Use Certification (DUC) may obtain ABCD Study data. ABCD Study data can be obtained by applying to the NDA directly here: https://nda.nih.gov/abcd/
